# Palmitate up-regulates laminin expression via ROS/integrin αvβ3 pathway in HLSECs

**DOI:** 10.18632/oncotarget.26937

**Published:** 2019-06-25

**Authors:** Qi Zhang, Luxia Jiang, Jing Yu, Limin Tian, Tiankang Guo, Baoshan Di, Jinxing Quan, Jing Feng, Jing Liu

**Affiliations:** ^1^Department of Endocrinology, Gansu Provincial Hospital, Lanzhou City 730000, Gansu Province, China; ^2^School of Basic Medical Sciences, Lanzhou University, Lanzhou City 730000, Gansu Province, China; ^3^Intensive Care Unit, Lanzhou University Second Hospital, Lanzhou City 730000, Gansu Province, China; ^4^Gansu University of Chinese Medicine, Lanzhou City 730000, Gansu Province, China; ^5^Emergency Department, Gansu Provincial Hospital, Lanzhou City 730000, Gansu Province, China; ^*^These authors contributed equally to this work

**Keywords:** human liver sinusoidal endothelial cells, integrin αvβ3, laminin

## Abstract

**Aims/Introduction:**

To investigate the roles of reactive oxygen species (ROS) and integrin αvβ3 in palmitate-induced laminin expression of human liver sinusoidal endothelial cells (HLSECs).

**Results:**

The protein expression of integrin αv, integrin β3 and laminin are increased by palmitate in HLSECs in a time- and dose-dependent manner. NAC, the ROS inhibitor, significantly inhibited the up-regulation of protein expression of integrin αv, integrin β3 and laminin by palmitate (*P* < 0.05). Palmitate markedly enhanced ROS formation (*P* < 0.05), which was not inhibited by LM609, the antibody of integrin αvβ3. Palmitate significantly increased laminin synthesis (*P* < 0.05), which was attenuated by LM609 and NAC (*P* < 0.05).

**Materials and Methods:**

HLSECs were treated with palmitate in the presence or absence of LM609 (10 μg/ml) or N-acetylcysteine (NAC) (2 mM). Expression of integrin αv, integrin β3 and laminin were measured by RT-PCR and Western blot. Immunocytochemistry were used for examining laminin expression. The generation of ROS was measured using the fluorescent signal 2',7' dichloro-fluorescein diacetate (DCFH-DA).

**Conclusions:**

The results suggested that palmitate increases laminin expression through ROS/integrin αv/β3 pathway.

## INTRODUCTION

With the increased incidence of diabetes, diabetic patients with non-alcoholic fatty liver disease (NAFLD) showed an increasing trend. It is reported that 28%–55% of type 2 diabetic patients had fatty liver [1], however, the specific pathogenesis is still unclear. Capillarization of liver sinusoidals plays an important role in NAFLD process, nevertheless, whether diabetes could accelerate the occurrence of sinusoidal capillaries rarely reported. Many study have demonstrated that oxidative stress and inflammatory response caused by lipid abnormalities are pathologic damage to the complications of diabetic angiopathy [2]. Palmitate, one of the most important saturated FFAs in human blood, induce oxidative stress and inflammatory response in endothelial cells. [3, 4].

HLSECs have unique and distinct characteristics with other vascular endothelial cells, cell surface sieves and basement membrane deficiencies. Hepatic sinusoid is not continuous sinusoid capillaries and has no basemen membrane [5]. The walls are composed of human liver sinusoidal endothelial cells (HLSECs), which are connected loosely and conducive to the exchange of material between sinusoidal blood and liver cells. The results show that NAFLD is associated with LSECs dysfunction. Researches had shown that sinusoidal capillarization can make the liver cells reduce the uptake of metabolites and oxygen, which result in the metabolism disorders, subsequently leading to NAFLD [6]. Couvelard et al. Have shown that absence of the basement membrane in normal liver may be a result of reduced expression of integrin and also indicates that the integrin is upregulated during liver fibrosis. Laminin is a major component of the basement membrane [7]. The integrin family is a group of transmembrane glycoproteins for ligands in the extracellular matrix. The vitronectin receptor, αvβ3 integrin, is highly expressed in endothelial cells of angiogenic vessels [8].

Although many studies explored the process of capillarization of liver sinusoids, the mechanism are still kept unknown. Few studies focused on the HLSECs respond to FFAs. On this basis, we investigate the effect and possible mechanism of palmitate on expressions of integrin αv, integrin β3 and laminin.

## RESULTS

### Palmitate increased ROS, integrin αv, integrin β3 and laminin expression in HLSECs

**Figure 1:**
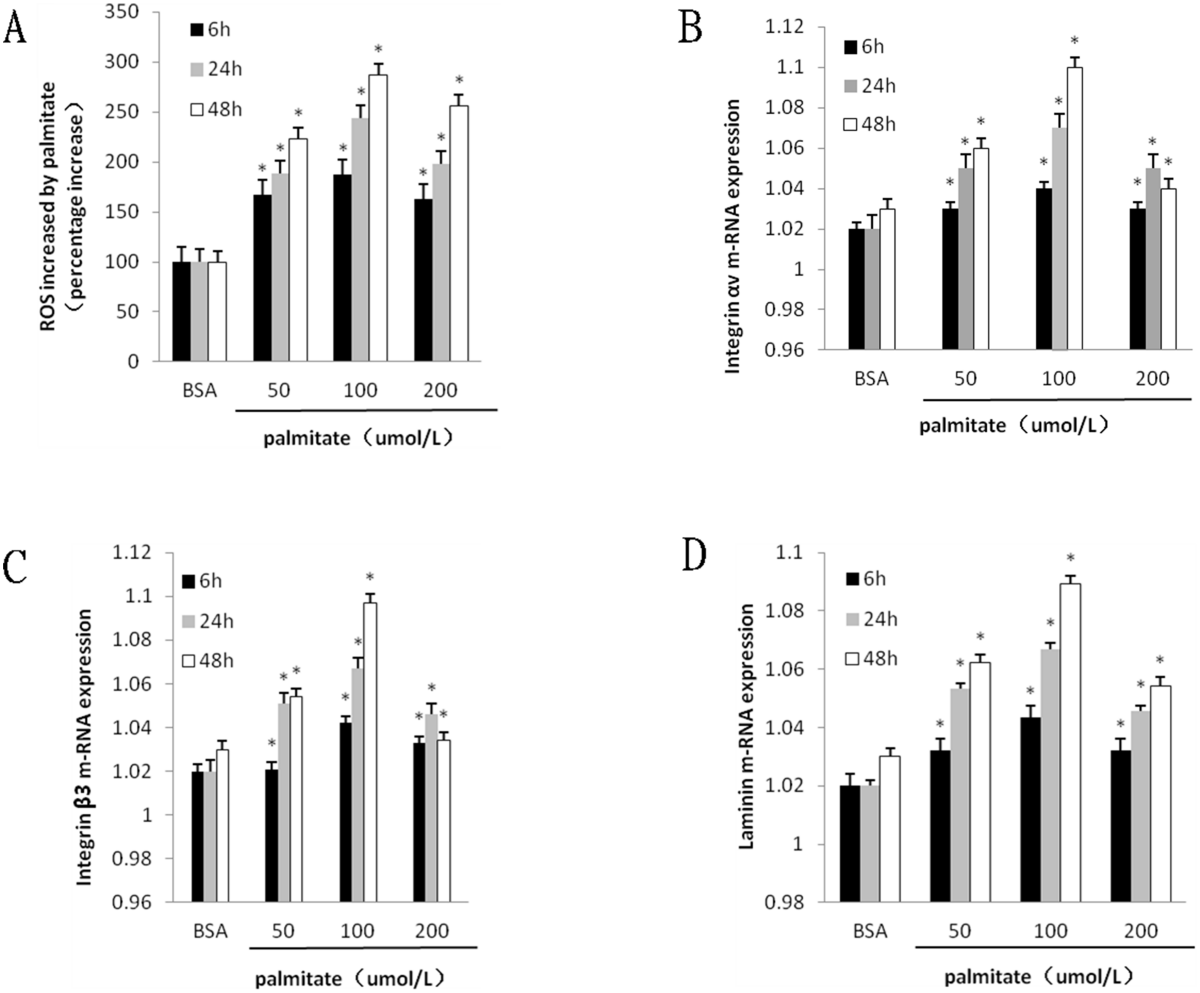
Palmitate induced ROS formation and the mRNA expression integrin αv, integrin β3 and laminin in HLSECs. Cells were cultured with BSA and palmitate 50 umol/L, 100 umol/L 200 umol/L for 6, 24 and 48 h respectively. (**A**) Statistical data of ROS expression by the fluorescent signal DCFH-DA. (**B**) Statistical data of integrin αv mRNA expression by Quantitative RT-PCR. (**C**) Statistical data of integrin β3 mRNA expression by Quantitative RT-PCR. (**D**) Statistical data of laminin mRNA expression by Quantitative RT-PCR Data are expressed as mean ± S.D. from three independent experiments, ^*^P < 0.05 vs control (BSA).

**Figure 2:**
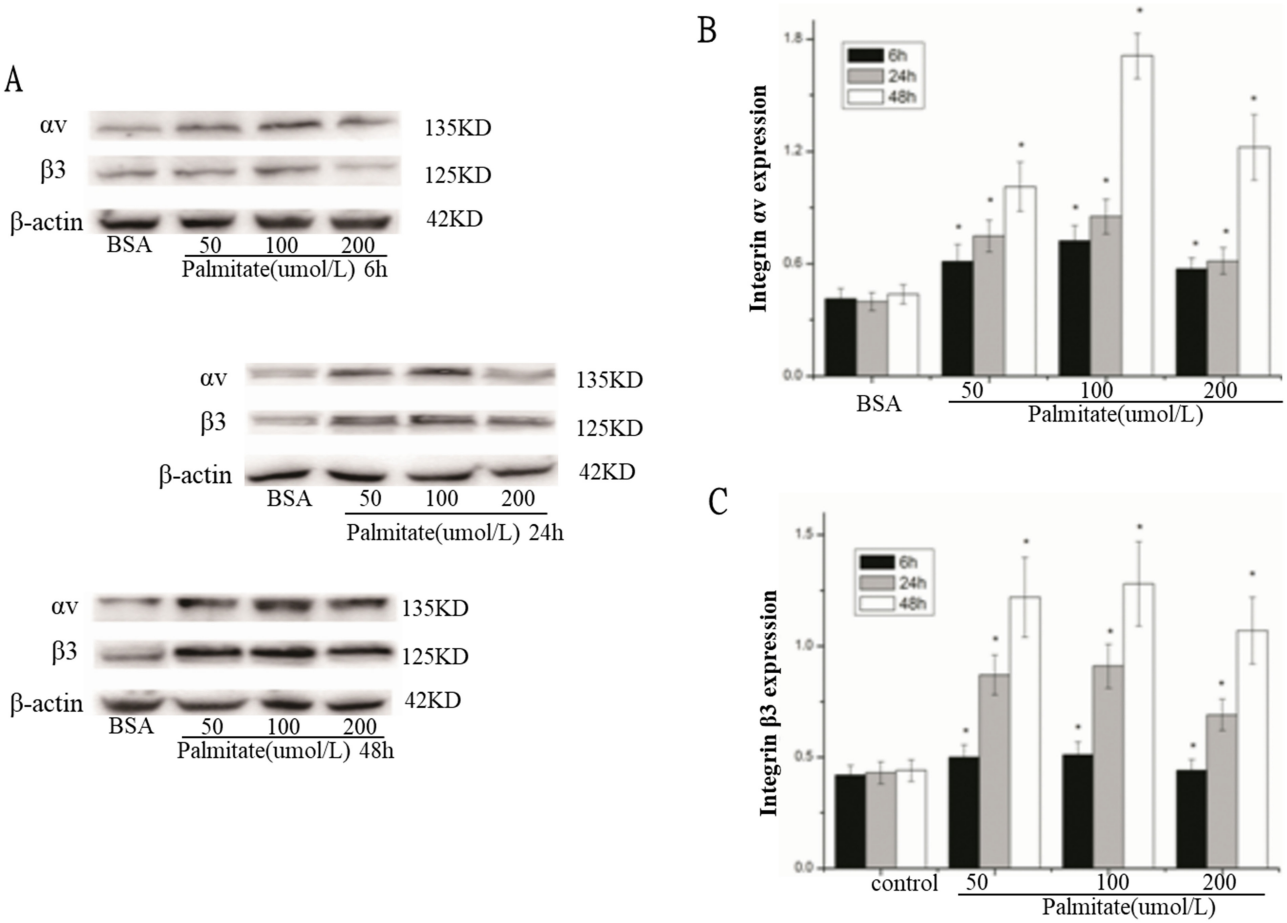
Palmitate increased the protein expression of integrin αv and integrin β3 in HLSECs. (**A**) Representative photograph of Western blot. (**B**) Statistical data of integrin αv expression. (**C**) Statistical data of integrin β3 expression. Results are expressed as mean ± S.D. from three independent experiments, ^*^P < 0.05 vs control (BSA).

To investigate the effect of palmitate on ROS, integrin αv, integrin β3 and laminin expression, HLSECs were treated with 50, 100 and 200 μmol/L palmitate for 6, 24 and 48 h respectively. The results revealed that both mRNA and protein expression of integrin αv, integrin β3 and laminin increased in response to palmitate in a time- and dose-dependent manner with exception of the data at 200 μmol/L palmitate (Figure 1B, 1C, 1D; Figure 2 and Figure 5, Figure 6). The generation of ROS measured using the fluorescent signal DCFH-DA was also increased in response to palmitate in a time- and dose-dependent manner (Figure 1A and Figure 4). In addition, expression of ROS, integrin αv, integrin β3 and laminin all peaked at 100 μmol/L palmitate for 48 h (integrin αv 4.3-fold vs control, integrin β3 2.9-fold vs control, *P <* 0.05). Therefore, 100 μmol/L palmitate for 48 h were used for all subsequent experiments.

**Figure 3:**
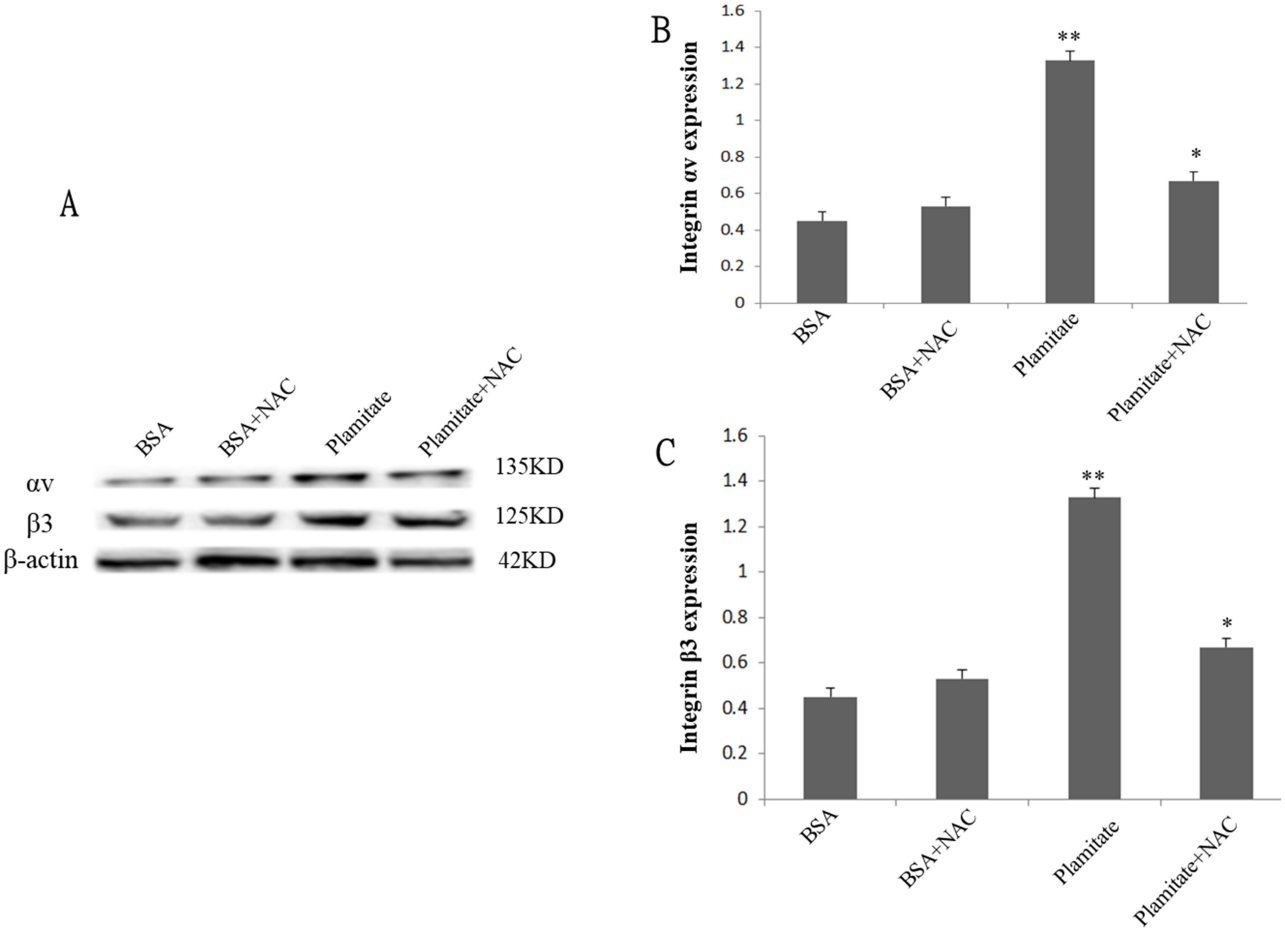
Effect of NAC (2 mM, 24 h) on integrin αv and integrin β3 expression. Cells were treated with 100 μmol/L palmitate for 48 h. (**A**) Representative photograph of Western blot. (**B**) Statistical data of integrin αv expression. (**C**) Statistical data of integrin β3 expression. Results are expressed as mean ± S.D. from three independent experiments, ^*^P < 0.05 vs control (BSA), ^**^P < 0.05 vs palmitate.

**Figure 4:**
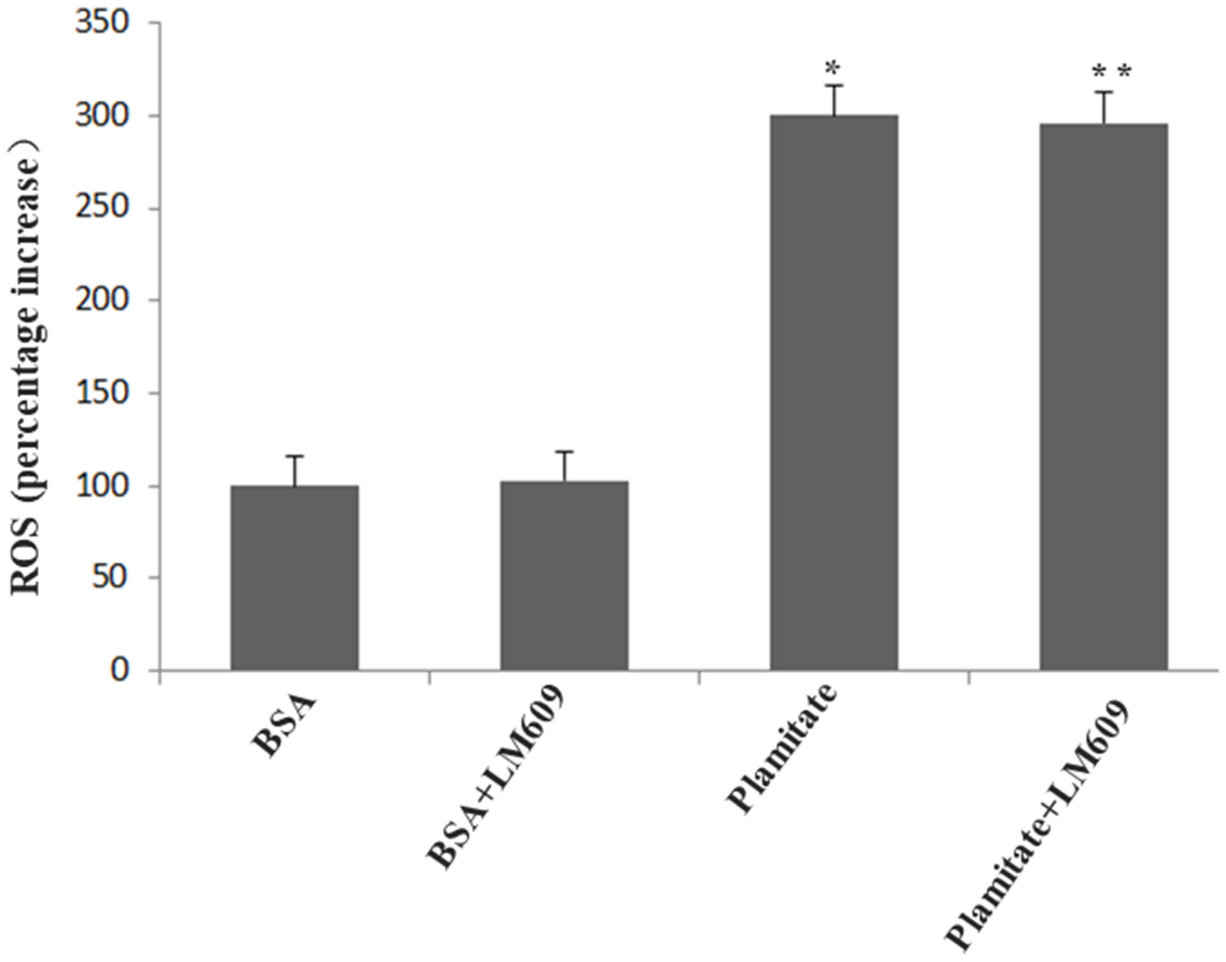
Palmitate induced ROS formation and effect of LM609 (10 μg/ml, 12 h) on ROS expression in HLSECs. Cells were treated with 100 μmol/L palmitate for 48 h. Results are expressed as mean ± S.D. from three independent experiments, ^*^P < 0.05 vs control (BSA).

**Figure 5:**
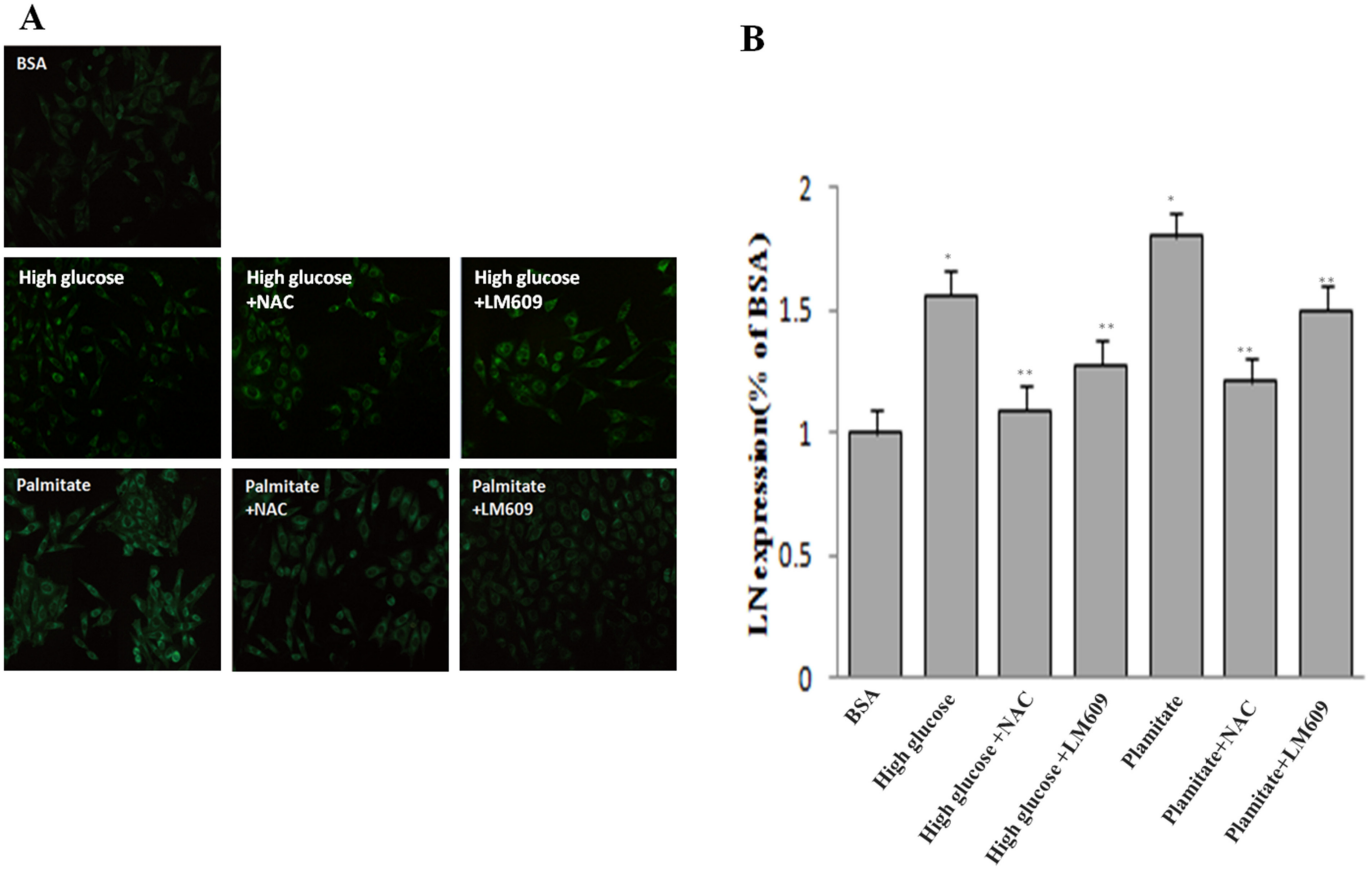
Effect of palmitate, NAC and LM609 on protein expression of laminin in HLSCEs. Cells were treated with 100 μmol/L palmitate for 48 h. (**A**) Representative photograph of Immunocytochemistry. (**B**) Statistical data of laminin expression. Results are expressed as mean ± S.D. from three independent experiments, ^*^P < 0.05 vs control (BSA), ^**^P < 0.05 vs palmitate.

**Figure 6:**
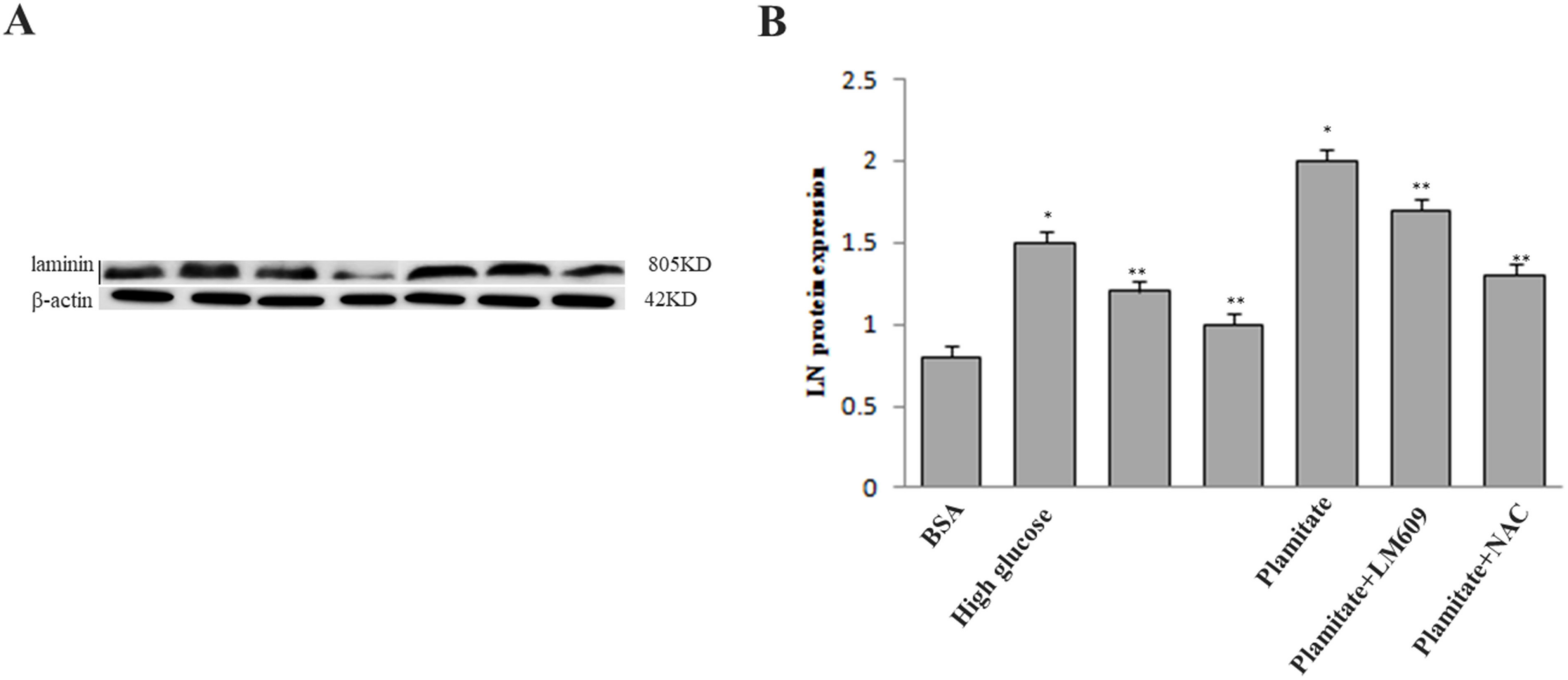
Effect of palmitate, NAC and LM609 on protein expression of laminin in HLSCEs. Cells were treated with 100 μmol/L palmitate for 48 h. (**A**) Representative photograph of Western-Blot. (**B**) Statistical data of laminin expression. Results are expressed as mean ± S.D. from three independent experiments, ^*^P < 0.05 vs control (BSA), ^**^P < 0.05 vs palmitate.

### NAC suppressed the expression of integrin αv, integrin β3 and laminin increased by palmitate

To explore the role of ROS in the up-regulation of integrin αv, integrin β3 and laminin by palmitate, NAC, the ROS inhibitor, was used. The results showed that NAC significantly inhibited the protein expression of integrin αv (Figure 3A and 3B), integrin β3 (Figure 3A and 3C) and laminin (Figure 5, Figure 6) in HLSECs treated with palmitate, in addition, laminin protein expression in palmitate group showed a greater extent than the high glucose control group (Figure 5, Figure 6), suggesting that palmitate increases the expression of integrin αv, integrin β3 and laminin, which is mediated by ROS formation.

### LM609 suppressed the expression of laminin increased by palmitate

To explore the role of integrin αvβ3 in the up-regulation of laminin by palmitate, LM609, the antibody of integrin αvβ3, was used. Both western blot and immunocytochemistry results revealed that LM609 significantly inhibited the protein expression of laminin in HLSECs treated with palmitate (Figure 5, Figure 6) however, had no effect on ROS production (Figure 4), suggesting that palmitate increased laminin expression through ROS mediated-integrin αvβ3 pathway.

### DISCUSSION

Chronic endothelial damage has been shown to have an important role in the development of diabetic angiopathy [9, 10]. Hepatic sinusoid endothelial dysfunction is an early event implicated in the progression to NAFLD, which also be a major cause of diabetic angiopathy, in addition, NAFLD is an independent predictor for diabetic macroangiopathy. Hepatic sinusoidal endothelial cells have no basement membranes but have many sinusoidal endothelial fenestrations (SEFs) in their membranes. collagen type IV, laminin, and fibronectin contribute on the formation of the liver basement membrane of the main material, in which laminin played a very important and irreplaceable role [11]. Sinusoidal endothelial capillaries caused by lipid disorders are multi-pathway multivariate, whereas up-regulation of laminin through ROS/integrin αvβ3 pathway is one of the ways that can not be ignored. Hyperlipidemia is an important factor in the development of liver diseases. Caballero etc. have demonstrated that the elevated circulating FFA levels cause insulin resistance, endothelial cells dysfunction and inflammation [12, 13]. HLSECs play a crucial role not only in clearance of waste products from the blood, regulation of pericyte contractility innate immune function, but also in their contribution and response to liver pathology. Chronic liver injury and liver cirrhosis are associated with angiogenic response with a formation of more classic vascular basement membrane [14–16]. Thus it is particularly important for this study.

In the present study, we reported for the first time that HLSECs express integrin αv, integrin β3 and laminin, which were increased by palmitate in a time- and dose-dependent manner to a greater extent than that under the high glucose condition. ROS formation and laminin synthesis were also found to be increased by palmitate. Furthermore, ROS antioxidant NAC significantly suppressed integrin αv, integrin β3 and laminin expression, and integrin αvβ3 antibody LM609 significantly prevented laminin expression but had no effect on ROS formation. These results suggested that ROS and integrin αvβ3 might get involved in the regulation of laminin by palmitate, subsequently resulting in the initiation and development of capillarization of the sinusoidals. It must be mentioned is that the expression of the protein instead starts to decline when the palmitic acid concentration is 200 μmol/L, which may be related to the high cytotoxicity of the palmitic acid.

Integrin family is a group of adhesion molecules which mediate cell-cell and cell-extracellular matrix adhesion. Integrin αvβ3 is expressed in high quantities on angiogenic endothelial cells, and facilitates cellular adhesion, proliferation, and migration [17, 18]. Integrin αvβ3 has also been shown to be a central role in angiogenesis and blockade of integrin αvβ3 activity using blocking antibodies or chemical inhibitors is known to disrupt angiogenesis in both *in vitro* and *in vivo* [19–21]. Anne Couvelard etc. have found that integrin expressed by sinusoidal endothelial cells in the normal liver is markedly altered during the process of capillarization. Integrin αvβ3 are present at low level along the sinusoidal wall and up-regulation of Integrin αvβ3 expression was found during the process of sinusoidal capillarization occurring in liver cirrhosis.

ROS plays a key role in the development of vascular disease. We observed the significant increase of ROS production in HLSECs treated with palmitate. Several studies suggest that ROS is an upstream regulatory factor [22], hyperglycemia increases ROS production via stimulation of mitochondrial respiration. The application of NAC reversed the effects of palmitate on the expression integrin αvβ3. Moreover, the shown results that decreased level of laminin in NAC group is more considerable than that in LM609 group demonstrated that oxygen stress reaction may be involved in Integrin αvβ3 up-regulation.

Our present study showed that laminin synthesis of cultured HLSECs induced by high lipid was also involved in increased expression of integrin αv and integrin β3. Jose V. Mpyano etc. have observed that expression of laminin-332 in MDCK cells is an autocrine response to endogenous TGF-β1 secretion and activation mediated by Integrin αvβ3 [23]. These results have suggested that integrin αvβ3 plays an important role in accelerating basement membrane formation which cause microangiopathy. The antibody LM609 to integrin αvβ3 significantly blocked the laminin synthesis in LSECs induced by palmitate, suggesting that integrin αvβ3 may play an important role as a downstream regulatory factor that mediates the synthesis of HLSEC laminin. In addition, neither inhibitor of ROS nor αvβ3 could completely blocked the expression of laminin in liver sinusoidal endothelial cells, indicating that pathways for regulating expression of laminin is not only the one we studied above but also other regulatory pathways need further investigation. Same results were reported in melanoma cells, which produce multiple laminin isoforms via integrin receptors [24]. Taken together, these findings suggested that increases in integrin αvβ3 expression may be the key events responsible for accelerated HLSECs laminin synthesis. High level of plasma lipid may be important pathogenetic factor that contribute to progressive capillarization of sinusoidals by the stimulation of Integrin αvβ3 expression.

In summary, we found in the present study that palmitate increased laminin expression in HLSECs through ROS mediated-integrin αvβ3 pathway, we will continue to confirm it *in vivo* experiments. Furthermore, these results could provide us with insights into NAFLD, which was associated with the up-regulation of integrin αvβ3 and oxidative stress as well as inflammatory response in HLSECs of Type 2 diabetes.

## MATERIALS AND METHODS

### Reagents

DMEM and foetal bovine serum (FBS) were purchased from Hyclone and Solarbio, Ltd (Beijing, China). Sodium palmitate and 2′,7′-dichlorofluorescein diacetate (DCFH-DA) were purchased from Sigma. Rabbit antibody to laminin was purchased from Abcam. Rabbit antibody to integrin αv and integrin β3 were purchased from Santa Cruz. Goat anti-rabbit HRP-conjugated secondary antibodies were purchased from ASGB-BIO, Ltd (Beijing, China). Monoclonal antibody LM609 to Integrin αvβ3 was obtained from Millipore. All other chemicals of analytical grade were purchased from commercial suppliers.

### Cell culture

Human liver tissue was obtained from a single 53-year-old Chinese woman undergoing resection for liver cysts under sterile conditions. Informed consent was obtained from the patient and this study was approved by the ethics research committee of The First Clinical College of Lanzhou University. HLSECs were isolated from 25 g of human liver tissue as described previously [25]. HLSECs were cultured in DMEM supplemented with 10% fetal bovine serum (FBS), as well as hepatocyte growth factor and vascular endothelial growth factor (both 10ng/ml) under standard cell culture conditions (humidified atmosphere with 5% CO_2_ at 37°C.). In addition, HLSECs were treated with palmitate (100 μM, 48 h) and high glucose (25 mM, 24 h) respectively in the presence or absence of LM609 (10 μg/ml, 12 h) or N-acetylcysteine (NAC) (2 mM, 24 h). The cell medium was changed every other day until confluence.

### Preparation of palmitate

Fatty acid was prepared according to previously reported methods [26, 27]. Briefly, the sodium salt of palmitate was dissolved in warm 50% ethanol at 70°C to make palmitate stock solution and then combined with 10% BSA while stirring at 37°C for 1 h. Further dissolution was performed in serum-containing medium at the final desired concentration.

### Measurement of intracellular ROS

The generation of reactive oxygen species (ROS) was measured using the fluorescent signal DCFH-DA. Cells cultured in 24-well plates were incubated with DMEM and harvested after 24 h. Cells with different treatment had been incubated with 10 μmol/L DCFH-DA for 30 min at 37°C in dark. DCFH-DA is converted by intracellular esterases to DCFH, which is oxidized into the high-fluorescence dichlorofluorescein (DCF) in the presence of a proper oxidant. HLSECs were washed with ice-cold PBS three times and placed in the dark. Subsequently, the DCF fluorescence was measured by Fluoroskan Ascent at 525 nm. The results were analyzed using the Cell Quest software.

### Quantitative RT-PCR

Total RNA was extracted with Trizol following the manufacturer’s instructions. RNA concentrations were determined using a spectrophotometer (Beckman Instruments, Fullerton, CA, USA). Approximately 2 μg RNA was reverse transcribed to cDNA using the Prime Script RT reagent kit. Quantitative RT-PCR (qRT-PCR) assays were performed using the LightCycler Real-Time PCR System (Roche 480). Samples were denatured at 95°C for 30 s followed by 40 PCR cycles of 95°C for 5 s, 60°C for 30 s and 72°C for 60 s. A melting curve was used to confirm the formation of the intended PCR products. The results are expressed as the fold difference relative to the level of -actin using the 2^-ΔΔCT^ method. Each reaction was performed in triplicate. Integrin av (NM 001106549.1) primers were: forward, 5-TTA TGC CAA AGA TGA CCC ACT-3 and reverse, 5-CGG GAC CTC CAA GAA GTA CTC-3. Integrin β3 (NM153720.1) primers were: forward, 5-TGG CAA GAT CAC CGG CAA GT-3 and reserve, 5-CAG TCC GAG TCA CAC ACG CA-3. Laminin (NM001374.2) primers were: forward, 5-GAC CCG TTC GGT TGT AAA T-3 and reverse, 5-GCC AGA CTC CAC CTC GTT A-3. β-actin (NM 001101.3) was used as an endogenous control with the following primers: forward, 5-TGG CAC CCA GCA CAA TGA A-3 and reverse, 5-CTA AGT CAT AGT CCG CCT AGA AGC A-3.

### Western blot analysis

Cells were lysed for 30 min at 4°C using lysis buffer. The extracted total protein concentrations were measured using the BCA Protein Assay Kit (Beyotime, China). Equal amounts of lysate proteins were loaded and separated by SDS-PAGE and transferred onto polyvinylidene fluoride membranes. After incubation in a blocking solution(5% non-fat milk, Sigma), the membranes were immunoblotted with primary anti-αv (1:200), anti-β3 (1:200, SantaCruz), anti-β-actin (1:1000) and laminin (1:500) overnight at 4°C. Membranes were washed and then incubated in a 1:1000 dilution of the specific secondary antibodies for 2 h at room temperature, and the membranes were washed with TBST for three times. The immunobands were detected using an enhanced chemiluminescence (ECL) western blot detection kit (Amersham Pharmacia Biotech). The film was canned using BIO RAD molecular imager with Image Lab Software, and the relative densities of protein bands were analyzed using the Image J Analyzer software. The density of each protein band was normalized to that of β-actin.

### Immunocytochemistry

Cells were treated as described in the figure legends. After stimulation, cells were fixed in paraformaldehyde for 15 min at room temperature and washed with PBS. Then cells were permeabilized 0.2% Triton-X-100 for 10 min and washed with PBS. Cells were blocked with 1% BSA 30 min. Samples were then incubated with anti-Laminin antibody at 1:100 dilution at 4°C overnight, and then incubated with Goat anti-rabbit HRP-conjugated secondary antibody at room temperature for 2 h. Images were observed by Olympus inverted fluorescence microscope.

### Statistical analysis

All data are expressed as mean ± S.D. from at least three independent experiments each performed in triplicate. Statistical comparisons between groups were made by analysis of one-way ANOVA. Analyses were done by the software SPSS 19.0. A value of *P <* 0.05 was considered statistically significant.
